# Toll-like receptor stimulation increases phagocytosis of *Cryptococcus neoformans* by microglial cells

**DOI:** 10.1186/1742-2094-10-71

**Published:** 2013-06-05

**Authors:** Sandra Redlich, Sandra Ribes, Sandra Schütze, Helmut Eiffert, Roland Nau

**Affiliations:** 1 Institute of Neuropathology, University Medical Center, Göttingen, Germany; 2 Department of Medical Microbiology, University Medical Center, Göttingen, Germany; 3 Department of Geriatrics, Ev. Krankenhaus Weende, Göttingen, Germany; 4 Department of Neuropathology, Georg-August-University, Göttingen, Robert-Koch Str. 40, Göttingen, 37075, Germany

**Keywords:** Toll-like receptor, Meningitis, Cryptococcosis, Microglia, Phagocytosis

## Abstract

**Background:**

Toll-Like receptors (TLRs) belong to the family of pattern-recognition receptors with a crucial function of recognising pathogen-associated molecular patterns (PAMPs). Cryptococcal meningitis is a potentially fatal disease with a high mortality and risk of neurological sequelae.

**Methods:**

We studied the ability of microglial cells to increase the phagocytosis of cryptococci after stimulation with agonists of TLR1/2, TLR3, TLR4 and TLR9.

**Results:**

Stimulation of murine microglial cells with these TLR agonists for 24 h increased the phagocytosis of encapsulated *Cryptococcus neoformans*. Stimulation increased the release of TNF-α, CXCL1 (KC), IL-6, IL-10 and MIP-2, which indicated the activation of microglial cells. Unstimulated and TLR agonist-stimulated MyD88-deficient cells showed a reduced ability to phagocytose cryptococci compared to their wild-type counterpart. Intracellular killing of cryptococci was also increased in TLR-stimulated cells compared to unstimulated microglial cells.

**Conclusion:**

Our observation suggests that stimulation of microglial cells by TLR agonists can increase the resistance of the brain against CNS infections caused by *Cryptococcus neoformans*. This may be of interest when an immunocompromised patient is unable to eliminate *Cryptococcus neoformans* despite antifungal therapy.

## Background

*Cryptococcus neoformans* (*C. neoformans*) is an encapsulated yeast which causes life-threatening infections in immunocompromised individuals, especially in patients with AIDS who are often unable to eliminate cryptococci completely from the cerebrospinal fluid (CSF) in an advanced stage of disease [1-3]. Cryptococcosis primarily affects these patients, but recently it has also been observed in immunocompetent individuals [4-8]. *C. neoformans* can be found worldwide particularly in bird guano, or in guano-contaminated soil. Upon inhalation of spores or yeast cells, it causes pulmonary infections. From the lung *C. neoformans* can disseminate into the skin and to the brain [9]. There it presents as meningoencephalitis or meningitis, which is fatal without antifungal therapy. The polysaccharide capsule is one of the most potent virulence factors of cryptococci and can inhibit phagocytosis [10]. After phagocytosis *C. neoformans* can survive and replicate in the acidic environment of the phagosome by increasing the pH [11].

Microglia, the resident phagocytes of the central nervous system (CNS), together with perivascular and meningeal macrophages constitute the first line of defense of the brain tissue in CNS infections [12,13]. In the resting state, they are scanning the CNS parenchyma by means of motile processes and will be activated when the microenvironment is changed. Microglial cells express Toll-like receptors (TLRs) which can identify pathogen-associated molecular patterns (PAMPs) and thereby play an important role as regulators of the innate immune response [14,15]. While bacterial lipoproteins and zymosan, a component of *Saccharomyces cerevisiae* (*S. cerevsiae*), are recognized by TLR2, lipopolysaccharide (LPS) from Gram-negative bacteria and glucuronoxylomannan, the major capsular polysaccharide of *C. neoformans*, are ligands of TLR4, and bacterial and also fungal DNA activates TLR9 [16-19]. Upon TLR stimulation, the ability of microglia to phagocytose Gram-positive and Gram-negative bacteria is increased [20,21]. We hypothesized that this mechanism could be a therapeutic option in fungal CNS infections. Therefore, we studied the phagocytosis of *C. neoformans* by microglial cells after TLR stimulation.

## Materials and methods

### Primary mouse microglial cell cultures

Primary mixed glial cultures were prepared from brains of newborn wild-type C57BL/6 mice (postnatal day 0, p0-p2), or myeloid differentiation factor (MyD)88-deficient mice (p0-p2) with the same genetic background [22]. After removal of the meninges, cells were treated with trypsin (Sigma-Aldrich, Taufkirchen, Germany) for 10 minutes to isolate the cells, and afterwards with DNAse (Sigma-Aldrich). After centrifugation cells were suspended in DMEM (Gibco, Karlsruhe, Germany) supplemented with 10% heat-inactivated FCS, 100 U/ml penicillin and 100 μg/ml streptomycin. Cells were plated at a density of two brains per T75 culture flask (Corning Costar, Wiesbaden, Germany) and incubated at 37°C with 5% CO_2_. After 10 to 14 days, microglial cells were isolated from the confluent astrocyte layer by shaking 200 times/minute for 30 minutes and plated in 96-well plates at a density of 50,000 cells per well.

### Microglia stimulation with TLR agonists Pam_3_CSK_4_, poly (I:C), LPS and CpG

Microglial cells from wild-type C57BL/6 and Myd88-deficient mice were exposed for 24 h to 0.1 μg/ml tripalmitoyl-S-glyceryl-cystein (Pam_3_CSK_4_; 910.5 Da; EMC Microcollections; Tübingen,Germany), 0.01 μg/ml LPS from *Escherichia coli* (*E. coli*) serotype O26:B6 (Sigma-Aldrich) or 1 μg/ml CPG oligodesoxynucleotide (ODN) 1668 (TCC ATG ACG TTG CTG ATG CT; molecular mass 6,383 Da, TIB Molbiol, Berlin, Germany), for TLR1/2, 4 and 9 stimulation, respectively. In experiments with MyD88-deficient mice, microglia were also exposed to 30 μg/ml of the TLR3 agonist polyinosine-polycytidylic acid (poly (I:C), 1.5 to 8 kb, InvivoGen, San Diego, CA, USA). TLR agonists were used at the lowest concentration that induced maximum stimulation as assessed by nitric oxide production [23]. A control group with unstimulated microglial cells was included in all experiments. For the measurement of cytokine release, supernatants from microglial cells stimulated for 24 h were collected and kept frozen at −20°C until assaying.

### Yeast strains and culture conditions

The encapsulated *C. neoformans* strain 11959 (ATCC 90112) was cultured in YPG medium (1% yeast extract,1% peptone, 2% glucose) at 30°C for 2 days. The cell suspension was mixed with glycerine and kept at −80°C. In each experiment, *C. neoformans* from the glycerine stock was grown on Sabouraud agar plates at 37°C. Colonies from the Sabouraud agar plate were resuspended in DMEM and counted in a Neubauer-hemocytometer to determine the concentration of fungi in the inoculum.

### Phagocytosis assay

After stimulation, microglial cells from either C57BL/6 wild-type or MyD88-deficient mice were co-incubated with the encapsulated *C. neoformans* resuspended in DMEM for 120 minutes at a number of 6 × 10^6^ colony-forming units (CFU)/well, with a ratio of approximately 120 yeast cells per phagocyte. After co-incubation with *C. neoformans*, microglial cells were washed with PBS and incubated with DMEM containing amphotericin B (2.5 μg/ml, Sigma-Aldrich) for 1 h to kill extracellular cryptococci. We confirmed amphotericin B activity by plating supernatants of each experiment after 1 h of incubation with amphotericin B. Thereafter, microglial cells were washed twice with PBS and lysed with distilled water. The ingested *C. neoformans* CFU were counted by quantitative plating of serial 1:10 dilutions on Sabouraud agar plates.

### Intracellular survival assay

TLR-stimulated or unstimulated microglial cells from wild-type C57BL/6 mice were incubated with *C. neoformans* for 120 minutes. Thereafter, cells were washed with PBS and incubated in DMEM containing amphotericin B (2.5 μg/ml) for up to 3 h to kill extracellular cryptococci. At different time points (60, 120 and 180 minutes), cells were washed with PBS and lysed with distilled water. The intracellular cryptococci were counted by quantitative plating of serial 1:10 dilutions on Sabouraud agar plates.

### Cytokine measurements

TNF-α, chemokine (C-X-C motif) ligand 1 (CXCL1) (also KC, or growth-regulated oncogene α (GROα)), IL-6, IL-10 and macrophage inflammatory protein (MIP)-2 were used to characterize microglial activation. DuoSet ELISA development kits (R&D Systems, Wiesbaden, Germany) were used for the cytokine measurements. The color reaction was quantified at 450 nm on a microplate reader (Bio-Rad, Munich, Germany).

### Measurement of cell viability

To measure the metabolic activity of viable cells, the WST-1 cell proliferation reagent (Roche Applied Science, Mannheim, Germany) was used. Microglial cells were incubated with 2.5 μg/ml amphotericin B for 3 h. Thereafter microglial cells were incubated with WST-1 for 3 h. Then, the produced formazan salt was measured by an increase of the optical density at 490 nm using a Genios multiplate reader (Tecan, Crailsheim, Germany). After 3 h of incubation, there was no toxic effect of amphotericin B on microglial cell viability (data not shown).

### Statistics

Statistical analysis and graphical presentation was performed by using GraphPad Prism 5 Software (GraphPad Software, San Diego, CA, USA). Data were expressed as the median with the 25% and 75% interquartile range, and were compared using the Kruskal-Wallis test followed by Dunn’s multiple comparison test for selected columns to correct for repeated testing. *P* <0.05 was considered statistically significant.

## Results

### Stimulation with Pam_3_CSK_4_, LPS or CpG increased the phagocytosis of *C. neoformans* by microglial cells

**Figure 1:**
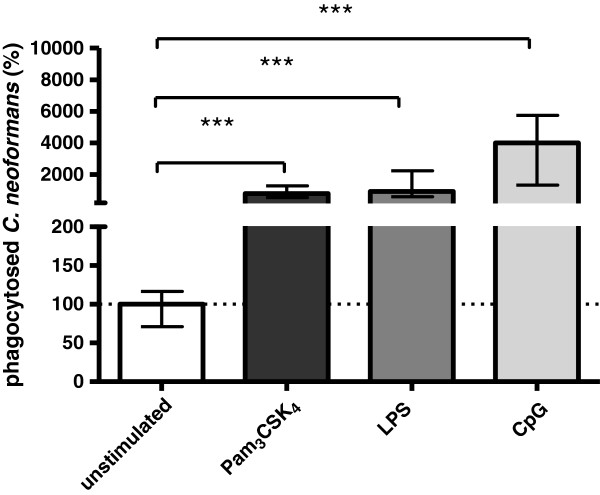
**Phagocytosis of *C. neoformans *by unstimulated and stimulated microglial cells.** Microglia were either unstimulated or stimulated with Pam_3_CSK_4_ 0.1 μg/ml, lipopolysaccharide (LPS) 0.01 μg/ml or CpG 1 μg/ml for 24 h (n ≥20 wells/group from five independent experiments). In each experiment, the mean number of bacteria ingested by the control group was considered to be 100%. Phagocytic rates of the stimulated groups are presented as percentages of phagocytosis by the unstimulated control group. Data are given as medians with interquartile ranges. Data were analyzed by Kruskal-Wallis test followed by Dunn’s multiple comparison test to correct for repeated testing; ^***^P<0.001.

The phagocytic rates of *C. neoformans* after 120 minutes of phagocytosis are shown in Figure 1. The number of intracellular cryptococci found in unstimulated microglia (12844 ± 6608 CFU/well expressed as mean ± SD) was considered to be 100%. Unstimulated cells ingested cryptococci at a lower rate compared to cells stimulated with TLR agonists. Pre-stimulation of microglial cells with different TLR agonists caused an increase in the uptake of cryptococci. Pre-stimulation with 0.1 μg/ml Pam_3_CSK_4_ and 0.01 μg/ml LPS increased the phagocytic rate approximately 15-fold (*P* <0.001) and 19-fold (*P* <0.001), respectively. The phagocytic rate after pre-stimulation with 1 μg/ml CpG was increased more than 36-fold (*P* <0.001). Additionally, we tested the extracellular amphotericin B activity in the cell culture medium after 1 h of incubation with amphotericin B by plating supernatants. The number of CFU was below the level of detection (10 CFU/ml) in each experiment.

### Enhanced phagocytosis required a functional MyD88 signaling cascade

**Figure 2:**
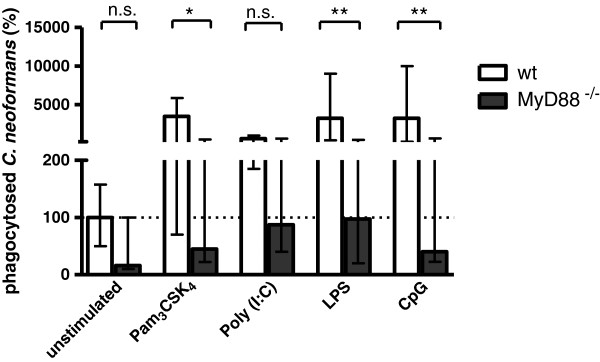
**Phagocytic rates of *C. neoformans *in stimulated wild**-**type and MyD88**-**deficient microglial cells after 120 minutes of phagocytosis.** Microglia were either unstimulated or stimulated with Pam_3_CSK_4_ 0.1 μg/ml, polyinosine-polycytidylic acid (poly (I:C)) 30 μg/ml, lipopolysaccharide (LPS) 0.01 μg/ml or CpG 1 μg/ml for 24 h. Phagocytosed cryptococci (colony-forming units (CFU)/well) were converted into percentage, and the median number of intracellular cryptococci from wild-type cells was considered to be 100% (n ≥12 wells/group from three independent experiments). Data are given as medians with interquartile ranges. Data were analyzed by Kruskal-Wallis test followed by Dunn’s multiple comparison test to correct for repeated testing; ^*^P <0.05, ^**^P <0.01; n.s., not significant; wt, wild-type.

Microglial cells from C57BL/6 wt and MyD88-deficient mice were stimulated with 0.1 μg/ml Pam_3_CSK_4_, 30 μg/ml poly (I:C), 0.01 μg/ml LPS and 1 μg/ml CpG. Phagocytosed cryptococci (CFU/well) were converted into percentage, and the median number of intracellular cryptococci found in unstimulated wild-type C57Bl/6 cells was considered to be 100% (Figure 2). Unstimulated MyD88-deficient microglia tended to phagocytose lower amounts of cryptococci than their wild-type counterpart (*P*-value not significant). After Pam_3_CSK_4,_ poly (I:C), LPS and CpG stimulation, MyD88-deficient microglial cells also had reduced ability to phagocytose *C. neoformans* compared to their wild-type counterparts (*P* <0.05 for Pam_3_CSK_4,_*P* <0.01 for LPS, and *P* < 0.01 for CpG)_._ The highest phagocytic rate of MyD88-deficient cells compared to the wild-type cells was seen after poly (I:C) stimulation (*P*-value not significant).

### Intracellular killing of *Cryptococcus neoformans* was enhanced after stimulation with Pam_3_CSK_4_, LPS and CpG

**Figure 3:**
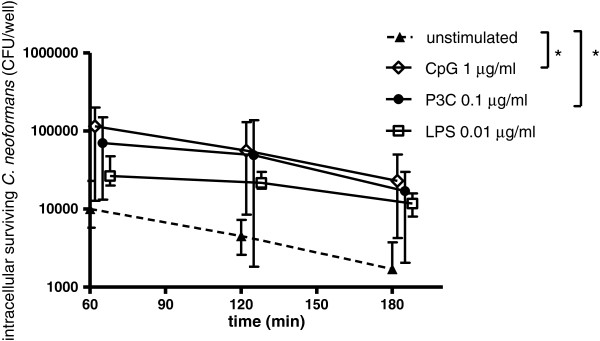
**Intracellular killing of *C. neoformans *by unstimulated and Toll-like receptor (TLR)**-**stimulated microglial C57BL**/**6 cells.** Intracellular killing was expressed as the number of cryptococci (median with interquartile ranges) recovered at the different time points (n ≥8 wells/group from two independent experiments). The absolute numbers of cryptococci killed after 3 h were higher in Pam_3_CSK_4_- and CpG-stimulated microglia than in unstimulated microglial cells; ^*^P <0.05.

The absolute numbers of intracellularly killed cryptococci after 3 h were higher in microglial cells after 24 h of Pam_3_CSK_4,_ LPS and CpG stimulation than in unstimulated cells. The median number of cryptococci killed by TLR-stimulated microglial cells was 53,000 CFU/well (Pam_3_CSK_4_), 14,750 CFU/well (LPS) and 92,000 CFU/well (CpG) compared to 8,300 CFU/well by unstimulated microglial cells (Figure 3). Stimulation of microglia with Pam_3_CSK_4_ or CpG resulted in significantly higher intracellular killing of cryptococci in comparison to unstimulated cells (*P* <0.05).

### Stimulation of microglia by TLR agonists induced TNF- α, CXCL1, IL-6, Il-10 and MIP-2 release

**Figure 4:**
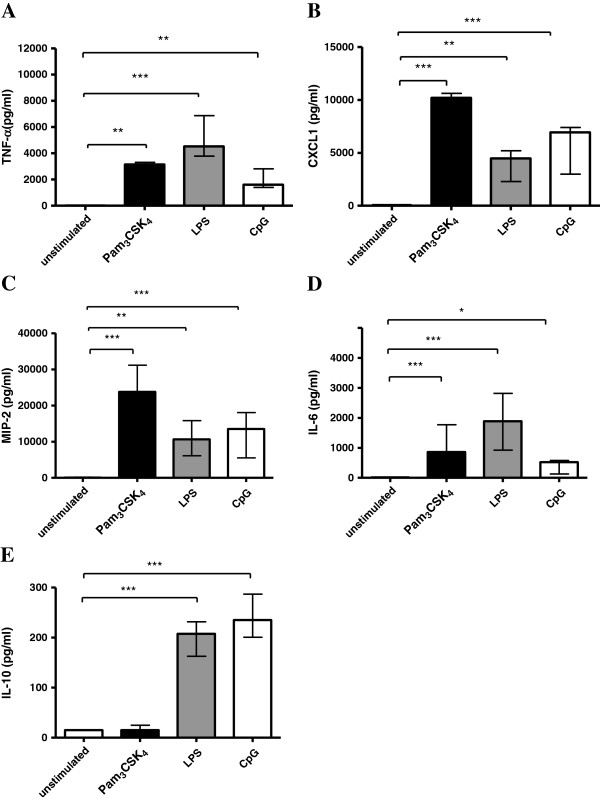
**Cytokine and Chemokine release of unstimulated and stimulated microglial cells.** Tumor necrosis factor (TNF)-α (**A**), chemokine (C-X-C motif) ligand 1 (CXCL1) (**B**), macrophage inflammatory protein (MIP)-2 (**C**), interleukin (IL)-6 (**D**) and IL-10 (**E**) concentrations (in pg/ml) in the supernatant of microglial cell cultures after 24 h of stimulation with 0.1 μg/ml Pam_3_CSK_4_, 0.01 μg/ml lipopolysaccharide (LPS) and 1 μg/ml CpG. Data are given as medians with interquartile ranges. Data were analyzed by Kruskal-Wallis test followed by Dunn’s multiple comparison test to correct for repeated testing; ^*^P <0.05, ^**^P <0.01, ^***^P <0.001.

To confirm microglial activation by the TLR agonists, we measured the concentration of TNF-α, CXCL1, IL-6, IL-10 and MIP-2 in the supernatants of microglial cultures after stimulation with Pam_3_CSK_4_, LPS and CpG (Figure 4). The limit of detection was 15 pg/ml for TNF-α, 62 pg/ml for CXCL1 and 31 pg/ml for IL-6, IL-10 and MIP-2. In the supernatants of unstimulated microglial cells after 24 h the median cytokine concentrations were below the detection limit for all cytokines and chemokines. Microglial stimulation with 0.1 μg/ml Pam_3_CSK_4_, 0.01 μg/ml LPS and 1 μg/ml CpG induced the release of high amounts of TNF-α, CXCL1, IL-6, IL-10 and MIP-2. Except for the IL-10 release after Pam_3_CSK_4_ stimulation, for all TLR agonists versus the control group the *P*-value was <0.05.

## Discussion

Current antifungal treatment of cryptococcal meningitis consists of amphotericin B plus flucytosine followed by fluconazole for a long period of time [24]. In clinical practice, AIDS patients show a reduced response to amphotericin B and flucytosine and often need lifelong prevention of relapses with fluconazole. For these immunocompromised individuals it would be highly beneficial to identify new therapeutic approaches helping the host to eliminate cryptococci from the CNS.

*In vitro* experiments have demonstrated that phagocytosis is dependent on the influence of opsonins [25]. TLRs play a crucial role in recognition of PAMPs and the pathogen-triggered inflammatory response [26]. We found that stimulation of microglial cells by different TLR agonists significantly increased the phagocytosis of fungi (present data) and bacteria [20,21]. This suggests that stimulation of microglial cells enhances the cellular innate immune response thereby increasing phagocytosis of invading bacteria or fungi and acts as an endogenous protective factor of the brain and myelon. The adaptor protein MyD88 is involved in the signaling cascade leading to the activation of nuclear factor-κB. All TLRs except TLR3 use the MyD88-dependent pathway. A mediator of the MyD88-independent pathway is the adaptor, TIR-domain-containing adapter-inducing interferon-β (TRIF), which relays signals of TLR3 and TLR4 [15]. We have shown that the increase of phagocytosis of *C. neoformans* by the different TLR agonists requires the functional MyD88 signaling cascade. After stimulation with Pam_3_CSK_4,_ LPS and CpG, the phagocytic rate of MyD88-deficient microglial cells was strongly diminished compared to wild-type microglia. Conversely the difference of phagocytosed cryptococci was not significant different in poly (I:C)-stimulated wild-type and MyD88-deficient microglia.

Intracellular survival, expulsion of the yeast by phagocytic cells, intracellular replication and cell-to-cell spread of *C. neoformans* play an important role in the pathogenesis of infection in immunosupressed patients [27,28]. Conversely, macrophages can phagocytose and kill *C. neoformans* in immunocompetent individuals [29]. When studying intracellular survival, intracellular replication of *C. neoformans* is a very important issue that has to be considered. It has been observed that cryptococci replicate faster intracellularly than extracellularly [30,31]. In our intracellular survival experiment over 3 h, we found a decrease of intracellular cryptococci in microglial cells and a higher absolute number of *C. neoformans* killed in Pam_3_CSK_4_-_,_ LPS- and CpG-stimulated microglial cells compared to unstimulated cells. Extracellular amphotericin B was necessary for the whole incubation time to avoid migration of viable cryptococci from the interior of the microglial cells to the medium, and subsequent extracellular growth. Microglia activated by TLR agonists produce proinflammatory cytokines (for example, TNF-α, IL-1 and IL-6) and nitric oxide, which can cause neuronal injury [32-36]. For this reason, the approach described here will have to be tested *in vivo* for efficacy and with respect to the possible induction of unintended neuronal injury. It may be necessary to design milder strategies for microglial activation in order to increase the infection resistance of the CNS without causing neuronal injury. In conclusion, our results suggest that the administration of TLR agonists is of potential therapeutic interest in the prevention and adjunctive treatment of *C. neoformans* meningitis and meningoencephalitis in high-risk groups such as patients with AIDS, or organ transplant recipients.

## Abbreviations

CFU: Colony forming units; CNS: Central nervous system; CSF: Cerebrospinal fluid; CXCL1: Chemokine (C-X-C motif) ligand 1; DMEM: Dulbecco’s modified Eagle’s medium; ELISA: Enzyme-linked immunosorbent assay; FCS: Fetal calf serum; GRO: Growth-regulated oncogene; IL: Interleukin; LPS: Lipopolysaccharide; MIP: Macrophage inflammatory protein; MyD: Myeloid differentiation factor; PAMP: Pathogen-associated molecular pattern; PBS: Phosphate-buffered saline; poly (I:C): Polyinosine-polycytidylic acid; TLR: Toll-like receptor; TNF-α: Tumor necrosis factor α; TRIF: TIR-domain-containing adapter-inducing interferon-β.

## Competing interests

The authors declare that they have no competing interests.

## Authors’ contribution

SRe performed the experiments and wrote the manuscript; SRi, SS and RN designed the study; HE provided the yeast strain. All authors read and approved the final version of the manuscript.

## References

[B1] Kobayashi M Murata K Hiroshi HO Tokura Y Cryptococcosis: long-lasting presence of fungi after successful treatment Acta Derm Venereol 2004 84 320 321 10.1080/00015550410025868 15339084

[B2] Casadevall A Spitzer ED Webb D Rinaldi MG Susceptibilities of serial Cryptococcus neoformans isolates from patients with recurrent cryptococcal meningitis to amphotericin B and fluconazole Antimicrob Agents Chemother 1993 37 1383 1386 10.1128/AAC.37.6.1383 8328793 PMC187974

[B3] Spitzer ED Spitzer SG Freundlich LF Casadevall A Persistence of initial infection in recurrent Cryptococcus neoformans meningitis Lancet 1993 341 595 596 10.1016/0140-6736(93)90354-J 8094831

[B4] Baddley JW Perfect JR Oster RA Larsen RA Pankey GA Henderson H Haas DW Kauffman CA Patel R Zaas AK Pappas PG Pulmonary cryptococcosis in patients without HIV infection: factors associated with disseminated disease Eur J Clin Microbiol Infect Dis 2008 27 937 943 10.1007/s10096-008-0529-z 18449582

[B5] Chen J Varma A Diaz MR Litvintseva AP Wollenberg KK Kwon-Chung KJ Cryptococcus neoformans strains and infection in apparently immunocompetent patients, China Emerg Infect Dis 2008 14 755 762 10.3201/eid1405.071312 18439357 PMC2600263

[B6] Zahra LV Azzopardi CM Scott G Cryptococcal meningitis in two apparently immunocompetent Maltese patients Mycoses 2004 47 168 173 10.1111/j.1439-0507.2004.00963.x 15078436

[B7] Kidd SE Hagen F Tscharke RL Huynh M Bartlett KH Fyfe M Macdougall L Boekhout T Kwon-Chung KJ Meyer W A rare genotype of Cryptococcus gattii caused the cryptococcosis outbreak on Vancouver Island (British Columbia, Canada) Proc Natl Acad Sci USA 2004 101 17258 17263 10.1073/pnas.0402981101 15572442 PMC535360

[B8] Suchitha S Sheeladevi CS Sunila R Manjunath GV Disseminated cryptococcosis in an immunocompetent patient: a case report Case Report Pathol 2012 201 652351 10.1155/2012/652351 PMC3420626 22953139

[B9] Liu TB Perlin DS Xue C Molecular mechanisms of cryptococcal meningitis Virulence 2012 3 173 181 22460646 10.4161/viru.18685 PMC3396696

[B10] Granger DL Perfect JR Durack DT Virulence of Cryptococcus neoformans. Regulation of capsule synthesis by carbon dioxide J Clin Invest 1985 76 508 516 10.1172/JCI112000 3928681 PMC423853

[B11] Levitz SM Nong SH Seetoo KF Harrison TS Speizer RA Simons ER Cryptococcus neoformans resides in an acidic phagolysosome of human macrophages Infect Immun 1999 67 885 890 9916104 10.1128/iai.67.2.885-890.1999 PMC96400

[B12] Hanisch UK Kettenmann H Microglia: active sensor and versatile effector cells in the normal and pathologic brain Nat Neurosci 2007 10 1387 1394 10.1038/nn1997 17965659

[B13] Nau R Bruck W Neuronal injury in bacterial meningitis: mechanisms and implications for therapy Trends Neurosci 2002 25 38 45 10.1016/S0166-2236(00)02024-5 11801337

[B14] Takeda K Akira S Toll receptors and pathogen resistance Cell Microbiol 2003 5 143 153 10.1046/j.1462-5822.2003.00264.x 12614458

[B15] Takeda K Kaisho T Akira S Toll-like receptors Annu Rev Immunol 2003 21 335 376 10.1146/annurev.immunol.21.120601.141126 12524386

[B16] Netea MG Ferwerda G van der Graaf CA Van der Meer JW Kullberg BJ Recognition of fungal pathogens by toll-like receptors Curr Pharm Des 2006 12 4195 4201 10.2174/138161206778743538 17100622

[B17] Underhill DM Macrophage recognition of zymosan particles J Endotoxin Res 2003 9 176 180 12831459 10.1179/096805103125001586

[B18] Gantner BN Simmons RM Canavera SJ Akira S Underhill DM Collaborative induction of inflammatory responses by dectin-1 and Toll-like receptor 2 J Exp Med 2003 197 1107 1117 10.1084/jem.20021787 12719479 PMC2193968

[B19] Yamamoto H Abe Y Miyazato A Tanno D Tanaka M Miyasaka T Ishii K Kawakami K Cryptococcus neoformans suppresses the activation of bone marrow-derived dendritic cells stimulated with its own DNA, but not with DNA from other fungi FEMS Immunol Med Microbiol 2011 63 363 372 10.1111/j.1574-695X.2011.00859.x 22092563

[B20] Ribes S Ebert S Regen T Agarwal A Tauber SC Czesnik D Spreer A Bunkowski S Eiffert H Hanisch UK Hammerschmidt S Nau R Toll-like receptor stimulation enhances phagocytosis and intracellular killing of nonencapsulated and encapsulated Streptococcus pneumoniae by murine microglia Infect Immun 2010 78 865 871 10.1128/IAI.01110-09 19933834 PMC2812218

[B21] Ribes S Ebert S Czesnik D Regen T Zeug A Bukowski S Mildner A Eiffert H Hanisch UK Hammerschmidt S Nau R Toll-like receptor prestimulation increases phagocytosis of Escherichia coli DH5alpha and Escherichia coli K1 strains by murine microglial cells Infect Immun 2009 77 557 564 10.1128/IAI.00903-08 18981243 PMC2612236

[B22] Regen T Van Rossum D Scheffel J Kastriti ME Revelo NH Prinz M Brück W Hanisch UK CD14 and TRIF govern distinct responsiveness and responses in mouse microglial TLR4 challenges by structural variants of LPS Brain Behav Immun 2011 25 957 970 10.1016/j.bbi.2010.10.009 20951794

[B23] Ebert S Gerber J Bader S Muhlhauser F Brechtel K Mitchell TJ Nau R Dose-dependent activation of microglial cells by Toll-like receptor agonists alone and in combination J Neuroimmunol 2005 159 87 96 10.1016/j.jneuroim.2004.10.005 15652406

[B24] Perfect JR Dismukes WE Dromer F Goldman DL Graybill JR Hamill RJ Harrison TS Larsen RA Lortholary O Nguyen MH Pappas PG Powderly WG Singh N Sobel JD Sorrell TC Clinical practice guidelines for the management of cryptococcal disease: 2010 update by the infectious diseases society of america Clin Infect Dis 2010 50 291 322 10.1086/649858 20047480 PMC5826644

[B25] McQuiston TJ Williamson PR Paradoxical roles of alveolar macrophages in the host response to Cryptococcus neoformans J Infect Chemother 2012 18 1 9 10.1007/s10156-011-0306-2 22045161 PMC4035814

[B26] Underhill DM Toll-like receptors: networking for success Eur J Immunol 2003 33 1767 1775 10.1002/eji.200324037 12811836

[B27] Alvarez M Casadevall A Cell-to-cell spread and massive vacuole formation after Cryptococcus neoformans infection of murine macrophages BMC Immunol 2007 8 16 10.1186/1471-2172-8-16 17705844 PMC1988836

[B28] Olszewski MA Zhang Y Huffnagle GB Mechanisms of cryptococcal virulence and persistence Future Microbiol 2010 5 1269 1288 10.2217/fmb.10.93 20722603

[B29] Levitz SM Cryptococcus neoformans: intracellular or extracellular? Trends Microbiol 2001 9 417 418 10.1016/S0966-842X(01)02137-0 11565559

[B30] Feldmesser M Kress Y Novikoff P Casadevall A Cryptococcus neoformans is a facultative intracellular pathogen in murine pulmonary infection Infect Immun 2000 68 4225 4237 10.1128/IAI.68.7.4225-4237.2000 10858240 PMC101732

[B31] Diamond RD Bennett JE Growth of Cryptococcus neoformans within human macrophages in vitro Infect Immun 1973 7 231 236 4697791 10.1128/iai.7.2.231-236.1973 PMC422665

[B32] Schutze S Loleit T Zeretzke M Bunkowski S Bruck W Ribes S Nau R Additive microglia-mediated neuronal injury caused by amyloid-beta and bacterial TLR agonists in murine neuron-microglia co-cultures quantified by an automated image analysis using cognition network technology J Alzheimers Dis 2012 31 651 657 22647259 10.3233/JAD-2012-120856

[B33] Iliev AI Stringaris AK Nau R Neumann H Neuronal injury mediated via stimulation of microglial toll-like receptor-9 (TLR9) FASEB J 2004 18 412 414 14688201 10.1096/fj.03-0670fje

[B34] Dawson VL Brahmbhatt HP Mong JA Dawson TM Expression of inducible nitric oxide synthase causes delayed neurotoxicity in primary mixed neuronal-glial cortical cultures Neuropharmacology 1994 33 1425 1430 10.1016/0028-3908(94)90045-0 7532825

[B35] Dawson TM Zhang J Dawson VL Snyder SH Nitric oxide: cellular regulation and neuronal injury Prog Brain Res 1994 103 365 369 7533914 10.1016/s0079-6123(08)61150-4

[B36] Chao CC Hu S Molitor TW Shaskan EG Peterson PK Activated microglia mediate neuronal cell injury via a nitric oxide mechanism J Immunol 1992 149 2736 2741 1383325

